# Fear and Anxiety in Pregnant Women During the COVID-19 Pandemic: A Systematic Review

**DOI:** 10.3389/ijph.2023.1605587

**Published:** 2023-02-24

**Authors:** Francisco Javier Muñoz-Vela, Luciano Rodríguez-Díaz, Juan Gómez-Salgado, Francisco Javier Fernández-Carrasco, Regina Allande-Cussó, Juana María Vázquez-Lara, Javier Fagundo-Rivera

**Affiliations:** ^1^ Nursing, University of Malaga, Málaga, Andalusia, Spain; ^2^ Regional University Hospital of Malaga, Málaga, Andalusia, Spain; ^3^ Nursing Department, Faculty of Health Sciences of Ceuta, University of Granada, Ceuta, Spain; ^4^ Sociology, Social Work and Public Health, Faculty of Labour Sciences, University of Huelva, Huelva, Spain; ^5^ Escuela de Posgrado, Universidad de Especialidades Espíritu Santo, Guayaquil, Ecuador; ^6^ Nursing and Physiotherapy Department, Faculty of Nursing, University of Cádiz, Cádiz, Spain; ^7^ Nursing, University of Seville, Seville, Andalucia, Spain; ^8^ Centro Universitario de Enfermería Cruz Roja, University of Seville, Seville, Andalucia, Spain

**Keywords:** anxiety, mental health, COVID-19, pregnant women, fear of childbirth

## Abstract

**Objectives:** The aim of this study was to explore the impact of the 2020–2022 pandemic on the levels of fear and anxiety in pregnant women and to identify risk and protective factors.

**Methods:** A systematic review was conducted. Electronic databases were consulted for studies published between January 2020 and August 2022. The methodological quality was assessed using a critical appraisal tool for non-randomised studies.

**Results:** Seventeen studies were included in the review. A high prevalence of levels of fear and anxiety were observed. Risk factors such as unplanned pregnancy, poor support from partners, or intolerance of uncertainty were identified for high levels of fear. Regarding anxiety, risk factors such as maternal age, social support, financial status, or concern about being able to maintain antenatal follow-ups were identified.

**Conclusion:** The COVID-19 pandemic had a significant impact on the mental health of pregnant women through increased levels of fear and anxiety. It has not been possible to establish a relationship between significant factors such as gestational age or health emergency control measures with high levels of fear or anxiety.

## Introduction

The year 2020 will go down in history as the year of the COVID-19 pandemic. This disease affected the global population and was of greater concern to certain vulnerable groups, such as healthcare professionals, who have had a higher level of exposure to the virus (1) and pregnant women, due to already existing concomitant diseases or life-threatening conditions (2). This population group’s cardiorespiratory and immune systems undergo substantial changes to accommodate the growing pregnancy. This fact characterises pregnancy as a period of particular vulnerability to infectious diseases (3-6). In this regard, there was differing research about the level of vulnerability of pregnant women compared to non-pregnant women during the first weeks of the pandemic. At the beginning, some research suggested that there was no increased threat for pregnant women during COVID-19 infection (7,8). However, more recent studies showed an increased risk of ICU admissions, hospitalisation (9) and, although vertical transmission is extremely rare (8,10), there does appear to be an increased risk for the development of pre-eclampsia, threatened preterm delivery, or low birth weight (11,12).

The fear of potential risk and lack of control caused by the COVID-19 pandemic has led to a perception of increased risk, according to the Perceived Risk Theory (13,14), as defined by other theories such as the Protection Motivation Theory (15) and the Health Belief Model (16), which propose two different aspects of risk perception: the subjective likelihood of contracting a disease or condition (perceived vulnerability) and the degree to which we are concerned (perceived severity) about the derived consequences. Given that the emergence of COVID-19 and its pandemic nature has exacerbated fears around the world, the situation of special vulnerability together with the uncertainty of its possible consequences on the unborn child make women during pregnancy a population group of special susceptibility to fear (17). Therefore, there is a need to assess these levels of anxiety and fear as attitudinal factors that may be relevant to adopt protective behaviours.

In this sense, many studies have focused on the impact of this COVID-19 pandemic on mental health (18,19). Also, previous studies have found that public health emergencies (e.g., 2003 SARS-CoV) triggered a range of emotional stress responses that involved high levels of anxiety and other negative emotions (20). In this line, during the SARS outbreak, higher levels of anxiety were associated with an increased likelihood of using at least five out of seven preventive measures (17). Further, a study developed during the 2009 influenza A (H1N1) pandemic showed how higher anxiety levels were significantly associated with lower use of more appropriate and consistent protective behaviours, but with a higher tendency to disinfect at home (21).

It has been shown that common psychological reactions to health crises are more likely to persist over time (22), and it has been argued that whenever fear or anxiety become chronic or irrational, they play a key role in the development of mental illnesses or psychological disorders such as stress or depression (23). Some research associates the presence of stress in pregnancy with alterations in the functioning of the newborn’s stress regulatory systems (24). Also, women’s experiences of fear of childbirth seemed to be related to their emotional wellbeing, stress symptoms, and impact on daily life. In addition, social circumstances, such as lack of social support, unemployment, and economic problems influence the likelihood of developing fear of childbirth (25,26). In this sense, it is suggested that those women for whom COVID-19 had a greater psychological impact were more likely to suffer from depression, with consequences both in the prenatal and postnatal period (27).

In this context, the aim of this review was to describe the impact of the COVID-19 pandemic on the levels of anxiety and fear in pregnant women, a group of particular vulnerability for the development of mental health problems.

## Methods

### Research Design

A literature review to assess the mental health of pregnant women during the pandemic was conducted using the systematic review format, following the criteria of the updated PRISMA 2020 (Preferred Reporting Items for Systematic Reviews and Meta-Analyses) guidelines (28). The implemented protocol was registered in the International Prospective Register of Systematic Reviews (PROSPERO) with code CRD42022355698.

#### Search Strategy

For the development of the research question, the standardised structure for formulating research questions, PECOT, has been followed (Table 1).

**TABLE 1:** PECOT format: Keywords (Spain, 2022).

P: Population	Pregnant woman
E: Exposure	COVID-19 disease
C: Comparison	Risk/protective factors
O: Outcome	Levels of fear and anxiety; prenatal care, risk factors and protection against prenatal levels of fear and anxiety
T: Timeframe	COVID-19 pandemic period

### Research Question

#### How Has COVID-19 Influenced Anxiety and Fear Levels During COVID-19 in Pregnant Women?

The following Medical Subject Headings (MeSH) descriptors were used to create the search string: pregnant woman, pregnancy, COVID-19, anxiety, and fear. In order to expand the search scope, free terms were added to the search using the Boolean operators AND and OR (Table 2).

**TABLE 2:** Terminology used in the search (MeSH terms) (Spain, 2022).


Pregnant woman OR Pregnancy OR pregnant women OR expecting mother
COVID-19
Anxiety
Fear

Table 3 shows the search process that was conducted on 2 September 2022 in the different databases (Pubmed, Scopus, Web of Science, and PsycInfo) using the different search strings and filtering from January 2020 to August 2022.

**TABLE 3:** Search strategy carried out in the different databases (Spain, 2022).

Database	Search strategy	Search date	Results
Pubmed	((pregnant women[Title/Abstract] OR pregnant woman[Title/Abstract] OR pregnancy[Title/Abstract] OR pregnant[Title/Abstract] OR expecting mother[Title/Abstract]) AND (COVID-19[Title/Abstract])) AND (Anxiety[Title/Abstract] AND fear[Title/Abstract])	2 September 2022	74
Scopus	(TITLE-ABS-KEY ("pregnant women" OR "pregnant woman" OR pregnancy OR pregnant OR "expecting mother") AND TITLE-ABS-KEY (COVID-19) AND TITLE-ABS-KEY (anxiety AND fear))	2 September 2022	132
Web Of Science	pregnant women OR pregnant woman OR pregnancy OR pregnant OR expecting mother (Topic) and COVID-19 (Topic) and Anxiety AND fear (Topic)	2 September 2022	139
PsycInfo	tiab(pregnant women OR pregnant woman OR pregnancy OR pregnant OR expecting mother) AND tiab(COVID-19) AND tiab(Anxiety AND fear)	2 September 2022	11
Total			356

### Selection Criteria

The following inclusion and exclusion criteria were used for the selection of articles.

#### Inclusion Criteria

- Research carried out between January 2020 and August 2022 (COVID-19 pandemic period).
- Type: meta-analysis, descriptive studies, correlational studies, cohort or case-control studies.
- Articles assessing the following indicators: levels of fear, levels of anxiety, comparison of levels of fear or anxiety before vs. during the COVID-19 pandemic, comparison according to prenatal care, risk and protective factors against prenatal levels of fear and anxiety.

#### Exclusion Criteria

- Articles of low methodological quality after assessment using a quality assessment tool.
- Articles on research involving pregnant women with pre-pregnancy mental illnesses.
- Articles assessing the indicators outlined in the inclusion criteria, but in non-prenatal periods.

### Data Collection and Extraction

The search was carried out independently by two reviewers using the agreed descriptors and the combination of the Boolean operators indicated in the search strategy. Subsequently, the articles were read and selected according to the inclusion criteria and applying the exclusion criteria. In case of disagreement over the inclusion of an article between the two co-authors, an online meeting was held with a third independent reviewer and, through a feedback process, a decision was made on whether or not to include the specific article.

### Methodological Quality Assessment

The assessment of methodological quality was performed independently by both reviewers using the critical appraisal tool of the Joanna Briggs Institute (JBI) tool for non-randomised studies at the University of Adelaide (29). This tool allows for the assessment of the methodology used in the research by identifying the absence of bias in its design, process, or analysis. In the present review, the version for cross-sectional quantitative studies consisting of eight items was used, and the cut-off point was set by consensus of both researchers at 6/8 to be considered eligible for inclusion.

## Results

In the previously mentioned databases and using the search strings in Table 3, a total of 356 articles were identified. After removing duplicate articles (165), a total of 191 articles remained eligible. Then, 113 articles were excluded after reading the title and abstract.

**FIGURE 1:**
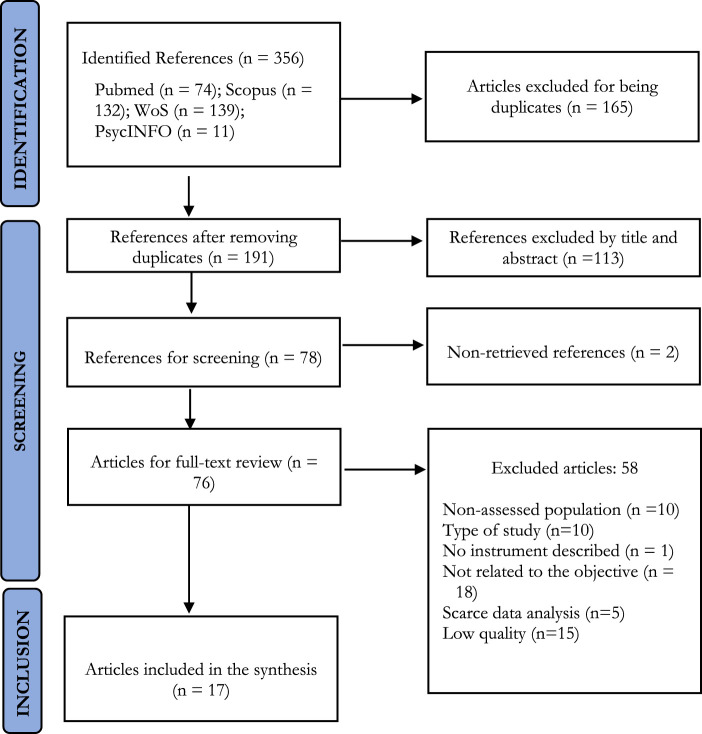
Identification of studies *via* databases (Spain, 2022).

Subsequently, 60 articles were eliminated after reading the full text for different reasons: type of study (*n* = 10), low methodological quality (*n* = 15), not related to the objective of the review (*n* = 18), no or little data analysis (*n* = 5), the study population was postpartum women (*n* = 9), no details of the instrument used (*n* = 1), or full text not available (*n* = 2). Figure 1 details the process followed for the identification, screening, and selection of the studies included in this review.

Finally, 17 articles were included in the review (30-46) that measured levels of fear or anxiety in pregnant women during the COVID-19 pandemic.

### Main Results

In order to synthesise the articles included in the review, Table 4 has been drawn up to display the main characteristics of the studies, including information on the author(s), location, main objective, type of research, sample, tools used, main results, and methodological quality.

**TABLE 4:** Characteristics of the studies included in the systematic review (Spain, 2022).

Study	Context	Study objective	Type of study	Participants and age	Instruments	Main results	JBI
García-Fernandez et al. (30)	Spain (September–December 2021)	To describe anxiety and stress of women in the first trimester of pregnancy in times of pandemic and its relationship with social support	Cross-sectional study	115 Mean 33.87	•Socio-demographic data	A high level of pregnancy-related anxiety was found among the study population, 78% according to the PRAQ-20 scale. Primiparous women showed higher levels of fear of childbirth compared to multiparous (12.13 ± 4.94 vs. 8.92 ± 4.32), although the reliability of the scale varied in nulliparous and multiparous, being 0.92 and 0.90, respectively. There was a significant correlation between stress and several variables: feelings about oneself, concern about the future, and concern for changes in oneself. The study suggests that high levels of stress were associated with high levels of social support, during the first trimester in conflict with similar studies	6/8
					•PRAQ-20		
					•MOS-S		
					•PSS		
Zilver et al. (31)	Netherlands (February 2019–January. 2020 and May–June 2020 and November–December 2020)	To assess pregnant women’s level of fear of childbirth during the first and second waves of COVID-19 compared with each other and with pregnant women before the pandemic	Cross-sectional study	2,197 Mean 31	•Socio-demographic data	The prevalence rate of fear of pregnancy (W-DEQA≥85) differed across groups ꭓ^2^ (2, *N* = 2,197) = 16.21, *p* < 0.01, being lower in the pre-pandemic group (*p* < 0.01). The trimester of pregnancy or previous psychological problems showed no modifying effect on the level of fear of pregnancy, *p* = 0.02 and *p* = 0.00 (*p* for interaction ≥0.13). Maternal age and parity showed a modifying effect (≤0.03 and <0.01, respectively)	7/8
					•W-DEQA		
Yeşilçinar et al. (32)	Turkey (May–July 2020)	To assess pregnant women’s levels of knowledge, fear, and anxiety during the COVID-19 outbreak	Cross-sectional study	184 Mean 29.31	•Socio-demographic data	The study showed a mean STAI score of 43.71 (9.44). The greatest concern about oneself was being infected with COVID-19 (60%) and the greatest concern in relation to pregnancy was transmission to the baby (60.6%). Having knowledge about COVID-19 according to the COVID-19 knowledge scale was significantly associated with higher scores on the STAI scale (r = 0.660; *p* = 0.34), and thus, higher levels of anxiety	6/8
					•STAI-I		
					• Questionnaire on knowledge, attitudes, and practice toward COVID-19		
Han et al. (33)	China (December 2021–April 2022)	To assess the prevalence of fear of childbirth and risk factors in pregnant women during the pandemic	Cross-sectional study	969 Mean 30.1	•Socio-demographic data	67.8% of the pregnant women showed symptoms of fear of childbirth (CAQ score ≥28), being severe in 4.0% of them (CAQ 52–64). The study shows that a significant difference between nulliparous and multiparous women in relation to the scores obtained in the CAQ and SCSQ scales (all *p* < 0.05). However, no difference was found between nulliparous and multiparous women in the IUS-12 scale. There was a positive correlation between fear of childbirth (CAQ) and negative coping styles (*r* = 0.375, *p* < 0.01) and intolerance of uncertainty (*r* = 0.397, *p* < 0.01), and a negative correlation between fear of childbirth (CAQ) and positive coping styles (*r* = −0.071, *p* < 0.05). Also, the correlation was statistically significant in the case of the negative coping styles subscale (SCSQ-N and IUS-12 (*r* = 0.404, *p* < 0.01)	7/8
					•CAQ		
					•IUS-12		
					•SCSQ		
Tuncer et al. (34)	Turkey (July–November. 2020)	To identify the relationship between anxiety and fear of childbirth among pregnant women during the COVID-19 pandemic	Cross-sectional study	261 Mean 27.76	•Socio-demographic data	44.8% of the pregnant women had a mid-level fear of childbirth. The study showed a mean childbirth fear score of 60.52 ± 23.75 (W-DEQ/Version A) and a mean anxiety score of 37.81 ± 9.28 (Spielberg’s State Anxiety Inventory), both levels of fear and anxiety higher than in pre-pandemic studies (46.4 ± 31.2 and 40.10 ± 4.24). The study indicates a relationship between anxiety levels and fear of childbirth (*r* = 0.511, *p* < 0.01)	6/8
					• Spielberg’s State Anxiety Inventory (STAI)		
					•W-EQ/Version A		
Makara-Studzińska et al. (35)	Poland (May–October 2020)	To assess the level of anxiety and its main determinants in women in the third trimester of pregnancy during the coronavirus pandemic	Cross-sectional study	315 Mean 28	•Socio-demographic data	According to the KLPII, 80% of the pregnant women showed fear of the duration of labour and only 38% showed control over the situation. The severity of labour-related anxiety was estimated with the LAQ scale. 33% showed high levels of tokophobia, and 26% very high levels. The study showed that level of education, place of residence, occupational activity, or location of delivery did not influence the levels of tokophobia. In terms of anxiety levels, according to STAI, 52.1% reported high levels of anxiety. A statistically significant relationship was identified between the time during the wave and the severity of situational anxiety, with October 2020 wave showing higher levels than May 2020 wave (*n* = 44 77.2% vs. *n* = 120, 46.5%, respectively) (Chi^2^ = 18.709; *p*-value=<0.001)	8/8
					•STAI		
					•KLPII		
Janik et al. (36)	Poland (April–July 2021)	To assess the level of COVID-19-related anxiety among pregnant women	Cross-sectional study	173 > 25<34 (76.30%)	•Socio-demographic data	Signs of generalised anxiety disorder were found in 23 pregnant women (13.3%), with a GAD-7 score >10 and a prevalence of anxiety symptoms of 62.5% (49% mild, 10% moderately severe, and 3.5% severe). Primiparas had higher SHAI scores (M = 14.45, Me = 14) and this was statistically significant (*p* = 0.031). However, the differences between primiparas and multiparas were not significant in the GAD-7 and STAI scales. Older pregnant women and with a higher educational level showed higher levels of anxiety (SHAI *p* = 0.019, M = 14.14, Me = 13; GAD-7 *p* = 0.006, M = 6.31, Me = 6). However, there were no statistically significant differences between hospitalised pregnant women during pregnancy and pregnant women not requiring hospitalisation	6/8
					•STAI		
					•GAD-7		
Shrestha et al. (37)	Nepal (July 2020)	To identify anxiety among pregnant women during the COVID-19 pandemic	Cross-sectional study	273	•Socio-demographic data	According to the scale used in the study, the largest group 91.57% (*n* = 250) showed a mild status regarding anxiety levels, 7.69% (*n* = 21) had mild to moderate anxiety, and 0.73% (*n* = 2) had moderate to severe anxiety. The sample according to areas of contagion were 3.1% red zone (highest incidence of contagion), 9.3% orange zone, 6.2% green zone, and 94% yellow zone	6/8
				Mean 27.2	•HAM-A		
Khamees et al. (38)	Egypt (November–December 2020)	To assess anxiety and depression in pregnant women during the pandemic	Cross-sectional study	120 Mean 27.8	•Socio-demographic data	The mean KUAS scores for nulliparous and multiparous women were 45.27 ± 10.78 and 47.28 ± 10.62. There was a significant association between the number of women reporting fear related to the COVID-19 pandemic and their scores on the scales (49.35 ± 9.64 and 14.97 ± 3.8 on KUAS and EDPS scores, respectively; *p*-value <0.001 each)	6/8
					•KUAS		
					•EPDS		
Akgor et al. (39)	Turkey (May 2020)	To determine the level of anxiety and depression and perspectives of pregnant women during the pandemic	Cross-sectional study	297	•Socio-demographic data	60.3% (*n* = 179) of pregnant women thought COVID-19 infection risk was higher in their babies compared to themselves, 51.5% (*n* = 153) were concerned about access to health services during the pandemic, and 66% were concerned about their antenatal follow-ups. Concern about not reaching the obstetrician for follow-ups was identified as a risk factor for anxiety (elevated HADS-A) (OR 1.42; 95% CI 1.18–3.16; *p* = 0.04). On the other hand, age (OR 1.41; 95% CI 0.33–2.87; *p* = 0.02) and concern about not reaching the obstetrician for follow-ups (OR 2.61; 95% CI 1.57–4.33; *p* = 0.001) showed a significant effect on depression. Fear of infection of the foetus during delivery revealed older age and having anxiety as the unique significant risk factors for anxiety. The study found that anxiety and depression scores of pregnant women with additional diseases were not higher than those of low-risk pregnant women	6/8
				Mean 27.64	•HADS		
Dymecka et al. (40)	Poland (March–May 2020)	To determine the relationship between fear of COVID-19, stress, and fear of childbirth	Cross-sectional study	262 Mean 28.40	•Socio-demographic data	Pearson’s r correlation analysis showed that there was a statistically significant, moderate, and positive relationship between the three tested variables: perceived stress, fear of COVID-19, and fear of childbirth. Fear of COVID-19 was a statistically significant mediator in the relationship between perceived stress and fear of childbirth (Pearson’s r correlation result: *M*-Mediator-Fear of coronavirus: M = 23.06; SD = 5.67; X = 0.26)	6/8
					•PSS-10		
					•FOC-6		
					•KLP II		
Nomura et al. (41)	Brazil (June–August 2020)	To study the prevalence of maternal anxiety in late pregnancy in the context of the COVID-19 outbreak in Brazil and to analyse its association with maternal knowledge and concerns about the pandemic	Multicentre cross-sectional study	1,662	•Socio-demographic data	According to the BAI scale, 13.9% of pregnant women in the last trimester had moderate anxiety, 9.6% had severe anxiety, and 22.4% mild anxiety (total: 45.9%). Different levels of anxiety were reported in different regions. Crude and adjusted analyses for confounding factors of the severity of maternal anxiety showed that the variable “cohabiting with a partner” (AOR = 0.53, 95% CI 0.38–0.75) was a protective factor for severe anxiety. The variables “secondary educational level” (AOR = 1.66, 95% CI 1.21–2.29), “alcohol consumption” (AOR = 3.5, 95% CI 1.94–6.14), and “having a family member diagnosed with COVID-19” (AOR = 1.88, 95% CI 1.11–3.16) were independent factors, significantly associated with moderate or severe maternal anxiety in late pregnancy	7/8
				Mean 28.02	•BAI		
Cigăran et al. (42)	Romania (May–October 2020)	To explore pregnant women’s perceptions of COVID-19 and the restrictions imposed and their experiences of care during the pandemic	Cross-sectional study	557 Mean not included	•Socio-demographic data	78.8%, *N* = 439 of the pregnant women were emotionally affected by the pandemic. Fear related to the possibility of having their pregnancy affected by the virus was dominant in 45.8% of the group. The study suggests that there is statistically significant evidence that pregnant women who stopped working reported panic and anxiety more frequently and those who continued working in the same conditions felt more frequent fear related to the possibility of having their pregnancy affected by the virus (*p* = 0.005, moderate effect size (Phi/Cramer’s V = 0.217/0.125). Fear was significantly associated with less confidence in the health system compared to people who said their emotional condition was not influenced by the pandemic, with a moderate effect size (*p* < 0.001 [df 4], [Phi/Cramer’s V = 0.220])	8/8
					•Ad hoc		
Ayaz et al. (43)	Turkey (April–May 2020 and prepandemic)	To compare the level of anxiety and depression in the same pregnant women before and during the COVID-19 pandemic	Cross-sectional study	63 Mean 30.35	•Socio-demographic data	The mean total IDAS II score was found to increase from 184.78 ± 49.67 (min: 109, max: 308) to 202.57 ± 52.90 (min: 104, max: 329) before and during the SARS-CoV-2 pandemic. During the SARS-CoV-2 pandemic, the difference in anxiety and depression between the periods was statistically significant (*p* < 0.001). Scores on the BAI scale suggest increased levels of anxiety: no anxiety (10–6); mild anxiety (31–24); moderate anxiety (20–25); severe anxiety (2–8)	6/8
					•IDAS II		
					• BAI (twice: pre-pandemic and during the pandemic		
Mappa et al. (44)	Italy (January–February 2020)	To assess the psychological impact of the COVID-19 pandemic on pregnant women during the period of peak virus spread and its relationship with pregnancy characteristics	Prospective observational study	178	•Socio-demographic data	The median value of STAI-T was 37% and 38.2% of the study group scored ≥40. The increase in STAI-S score is related to pre-existing anxiety states as measured by the STAI-T subscale. 83 participants (46.6% 95%CI 39.4–53.9) showed fear about the possible consequences of COVID-19 to the foetal structure. 116 participants (65.2% 95% CI 57.9–71.7) were concerned about growth, and 91 about preterm birth. Higher educational level was associated with higher values on the STAI-S subscale (*p* = 0.004), but not on the STAI-T subscale (*p* = 0.158)	6/8
				Mean 33	•STAI		
Fan et al. (45)	China (July 2020–July 2021)	To explore factors associated with depression and COVID-19-related fear among pregnant and new mothers	Cross-sectional study	3,027 Mean 30	•Socio-demographic data	Approximately 17.2% of the participants had depression, and 14.4% and 20.0% of the pregnant participants and postpartum women had depression, respectively (*p* < 0.001). The mean level of fear was M = 24.6; SD = 5.9; *p* < 0.001. A positive relationship was found between COVID-19-related fear and depression (*β* = 3.09; 95% CI = 2.57–3.62) and an inversely proportional relationship between fear and knowledge about how to prevent infection. Participants with greater knowledge about COVID-19 were less likely to have depression (OR = 0.91; 95% CI = 0.87–1.04)	6/8
					•PHQ-9		
					•Ad Hoc Fear Scale		
Kajdy et al. (46)	Multinational (May 2020–February 2021)	To assess risk factors for anxiety and depression among women prenatally during the COVID-19 pandemic, to compare differences in anxiety and depression scores between middle- and high-income economies, to assess the relationship between pandemic status and pregnant women’s mental health	Multinational, quantitative, cross-sectional, study	7,102 Mean 31.91	•GAD-7	Pregnant women with a high socioeconomic status showed higher PHQ-9 (0.18 SD, *p* < 0.001) and GAD-7 (0.08 SD, *p* = 0.005) scores than those of middle socioeconomic level. Risk factors for high PHQ-9 scores were previous mental problems and feeling worried about restrictions, and for GAD-7 these were pregnancy complications, fear of contagion of the newborn, and worry about financial status. Pregnant women of middle and high socioeconomic status showed the same sources of fear and burdens. The main reported source of fear and concern was restrictions in accompaniment during the pandemic. The main fear and concern in relation to the newborn were the infection and complications derived from COVID-19. In both economic cases, comfortable financial status, support from partner, family members, and maternal age were protective factors, with lower scores on GAD-7. Pregnant women of middle socioeconomic level showed higher levels of anxiety and depression about birth restrictions than those of high economic level	7/8
					•PHQ-9		

The origins of the different studies were varied: four of them had been carried out in Turkey (32,34,39,43); three in Poland (35,36,40); two in China (33,45); and the following countries were represented with one study each: Spain (30); Romania (42); Brazil (41); Egypt (38); Nepal (37); the Netherlands (31); and Italy (44). Finally, one multinational study was also included (46).

By way of summary, all studies included the pregnant female population in the antenatal period during the COVID-19 pandemic, which is the focus of the present study. However, two of the studies also included women during the pre-pandemic period (31,43) and one study also included women during the postpartum period (45).

Of the total of 17 studies included in the analysis, 15 were quantitative cross-sectional studies (30-45) and 2 were quantitative multicentre cross-sectional studies (41,46).

Assessing the levels of fear and anxiety in pregnant women was the main objective of this review and both variables were identified in 7 (31-34,40,42,45) and 13 (30,32,34-39,41-44,46) studies, respectively. Other factors related to the psychological impact that the COVID-19 pandemic had on the pregnant women were: stress (30,40) or depression (38,39,43,45,46). Both fear and anxiety were assessed using different measurement tools: fear was assessed using, W-DEQ-A (34), FOC-6 (40), and *ad hoc* Fear scale (42,45). On the other hand, anxiety was assessed with STAI (32,35,36,44), GAD-7 (36,46), PRAQ-20 (30), KLP-II (35,40), HAM-A (37), Kuas, Spielberg’s State Anxiety Inventory (34), HADS (39), BAI (41), and IDAS II (43). Lastly, the PSS (30) and PSS-10 (40) were used to measure stress.

In addition, other factors that were assessed in the different studies required the use of other scales such as the Social Support MOS-S (33), the Questionnaire on Knowledge, Attitude, and Practices toward COVID-19 (32), the CAQ for engaged behaviour (33), the IUS-12 for intolerance of uncertainty (33), the SCS-Q for coping skills (33), the EPDS for postpartum depression (38), and the PHQ-9 for the assessment of depression (45,46).

In relation to the sample collection period, thirteen studies were conducted in 2020 (31–46). Of these, two had also collected data in 2021 (45,46) and one study had collected pre-pandemic period data (43); five studies had collected data during 2021 (30,33,36,45,46) and one study included data from 2022 (33).

After the initial discussion on the inclusion of articles, a consensus was reached on 17 studies. To these, one article was added though doubts arose about its inclusion, leaving the decision to the discretion of a third reviewer. It was finally excluded it as it did not assess fear or anxiety but other mental health disorders.

Among the studies analysed, the methodological and quality assessment showed 11 studies with a score of 6, which was the minimum required in the selection criteria. In addition, 4 studies obtained a score of 7, and 2 studies a maximum score of 8.

### Level of Fear

Fear levels in pregnancy have been studied by many researchers in the pre-pandemic period as pregnant women are considered a high-risk vulnerable group compared to a non-pregnant population due to both physical and psychological changes that occur during pregnancy.

However, to date, no clear definition of fear in pregnant women has been identified. Fear of childbirth (FOC) seems to be used as a general term, which has led to considerable heterogeneity around the presence of many types of anxiety and fear among women during pregnancy. Thus, it can be concluded that FOC seems to be an expression for different emotional difficulties during pregnancy or in the postpartum period.

In this context, and given the lack of knowledge about women’s fear of the unpredictability of COVID-19, the unavailability of reliable and valid scales to assess fear in a pandemic context, the lack of specific questions related to fear of COVID-19 in the different scales available and, consequently, the lack of discriminatory properties of these scales, the results of the present study are clinically relevant, as it has been determined to assess the association between the general fear of pregnant women during pregnancy and childbirth and a limited number of variables related to gestation during the COVID-19 pandemic period.

One of the studies, conducted by Zilver et al. (31), showed lower levels of fear of childbirth in the first months of the pandemic compared to the pre-pandemic period. Possible explanations for these results include: the study included only nulliparous women; less stress and pressure on daily life due to the recent implementation of teleworking in the first months of the pandemic; better sleep levels; increased physical exercise; greater family support; optimisation of sleeping hours. However, the other six studies assessing fear in pregnancy during the pandemic (32-34,40,42,45) showed opposing results to the ones found by Zilver et al. (31), but showed similar results among them. Makara-Studzińska et al. (35) suggested that 80% of pregnant women were fearful of the length of labour, and Tuncer et al. (34) indicated that 44.8% of pregnant women had an intermediate level of fear during the pandemic. Khamees et al. (38) indicated that 77.5% of pregnant women thought that the pandemic would be a threat to their health. Cigaran et al. (42) reported that 45% of pregnant women showed fear related to the possibility that the pregnancy would be affected by the coronavirus infection, and the study by Fan et al. (45) reported a prevalence of fear of childbirth during the pandemic of 67.8%, i.e., more than half of the pregnant women had fear of childbirth during the pandemic. In this same study, a prevalence of 67.8% was reported; more than half of the participants indicated that thoughts of COVID-19 frightened them and that they felt nervous when they thought about it (45). Yesilcinar et al. (32) reported, between May and July 2020, that 77.6% of pregnant women had fear of coronavirus transmission and 67.6% stated that the COVID-19 pandemic had had an impact on mental health.

Among the main factors associated with fear during the pandemic, parity stands out, with Han et al. (33) showing that there was a significant difference between nulliparous and multiparous women in levels of fear of pregnancy during the last months of the pandemic, suggesting nulliparity as a main risk factor along with others such as unplanned pregnancy, poor support from partners, negative behaviours, or fear of the unknown. In the same vein, Makara-Studzinska et al. (35) observed that age had an influence on the severity of tokophobia or fear of pregnancy, indicating that older women showed lower levels than younger women. They also suggested that financial status correlated with levels of tokophobia, being higher in women with lower financial status.

### Level of Anxiety

Anxiety is defined as an unpleasant and unclear emotional state related to the anticipation of external danger or originating from within the body (35). A total of eight different tools or scales were used to measure anxiety levels in the different studies included in the present review, which may justify the existence of such disparate results due to a bias in their quantification.

Garcia Fernandez et al. (30) found a pregnancy-related anxiety level of 78%, which are very high figures in contrast to other studies, such as the one by Nomura et al. (41) with a sample of 1,662 pregnant women in the last trimester of pregnancy of which 13.9% had moderate anxiety levels and 9.6% had severe anxiety, also reporting different levels of anxiety in different regions. There is, however, a wide variability in the prevalence of anxiety in the different samples of the studies conducted during the COVID-19 pandemic. This could be explained by the different phases through which the pandemic has evolved, by the impact of different disease control and prevention measures in each region, by the differences in anxiety levels in the phases of the gestational period, or by the different pre-existing anxiety levels between nulliparous and multiparous women. In fact, Nomura et al. (41), as well as Garcia Fernandez et al. (30), with a sample of 1,662 and according to the BAI scale, found different levels of anxiety in different regions of Brazil. Janik et al. (36) found a prevalence in anxiety symptoms of 62.5% in the months of April to July 2021 and primiparous obtained higher scores in the SHAI scale, being statistically significant (*p* = 0.031). Makara-Studzinska et al. (35), between May and October 2020, identified a statistically significant influence of the time during the wave on the severity of situational anxiety, with the October 2020 wave yielding higher levels of anxiety than the May 2020 wave.

Along these lines, the different studies included in the review have identified a series of factors associated with higher levels of anxiety: parity, training or educational level, maternal age 4,036, greater knowledge about COVID-19 (32), concern about maintaining follow-up of the pregnancy (39), loneliness or lack of a companion during pregnancy, alcohol consumption, and having a relative diagnosed with COVID-19 (41). All these risk factors have increased anxiety levels during the pandemic and there is no doubt that pregnant women have a series of needs in terms of information, family, and work based on their own personal or health characteristics, needs which are increased by pandemic situations and that can affect the wellbeing of both the woman and the foetus. In this sense, Cigaran et al. (42) reported that 78% of pregnant women were emotionally affected by the pandemic. Fear related to the possibility of the pregnancy being affected by the virus was dominant in 45.8% of pregnant women, and women who stopped working reported panic and anxiety more frequently than those who continued working under the same conditions, with this difference being statistically significant. The most common personal concern related to the pandemic was risk to the foetus (39), and it was identified that concern about maintaining antenatal follow-up was a risk factor for both anxiety and depression.

While some factors were shown to predispose to increased levels of fear or anxiety in pregnant women, there are other factors that may be considered protective, such as living with a partner (41), having a comfortable financial status, family support, or maternal age (46).

## Discussion

Through the present review, levels of fear and anxiety in pregnant women during the COVID-19 pandemic have been made visible and possible risk or resilience factors have been identified.

The analysed studies showed a high prevalence of fear levels, with between 44.8% and 80% of pregnant women showing intermediate to high levels of fear during the COVID-19 pandemic. The manifestation of fear levels is common during pregnancy, with women experiencing some level of fear during pregnancy and in the postpartum period, around 14% according to some pre-pandemic studies (39,47). Thus, the impact of the global COVID-19 pandemic has clearly contributed to higher levels of fear (31,34,48) despite the different characteristics of the studied populations: gestational age, regions, maternal age, parity, the use of different measurement tools and study methods, problems with definitions of fear in pregnant women, or even the different phases of the pandemic from its onset in late 2019 to the present day when, for different reasons, this disease is already part of our lives. These are some of the factors that may have influenced the wide variability in the prevalence of gestational fear during the pandemic.

According to a systematic review, the prevalence of fear in pregnancy was 14% before the pandemic (47), and other studies correlated mild and high levels of fear of childbirth with prolonged labour, caesarean section, increased use of epidural analgesia, antenatal and postpartum depression, or axiety (49-53). The fear of being infected by a virus whose consequences were scientifically unknown or without a specific treatment, together with social distancing as the main measure to avoid spread of the virus, contributed to social isolation, isolation from other family members, from friends, from work colleagues, or from the health system itself. In this line, a series of factors associated with higher levels of fear have been identified in the analysed studies, such as nulliparity (30,31,46), unplanned pregnancy, poor support from partners (46), maternal age (31,46), financial status (46), or negative behaviours (33). All of these risk factors have increased their impact during the COVID-19 epidemic and, as reported by Cigăran et al. (42), in 45.8% of women fear is related to the possibility of the pregnancy being affected by the virus.

There are a number of factors with no clear evidence of a modifying effect. Pregnancy trimester or gestational age, educational level, place of residence, professional activity, or place of maternity care do not appear to influence the levels of fear (31,35). In relation to whether educational level may affect the prevalence of fear, the study by Makara-Studzińska et al. (35) in Poland showed no modifying effect in this regard. However, Fan et al. (45) in China reported that knowledge about infection prevention was statistically significantly associated with lower levels of fear, with the level of knowledge reported by the sample being moderate. Also, the study by Yeşilçinar et al. (32) stated that having greater knowledge about COVID-19 resulted in higher levels of anxiety. These, *a priori*, contradictory results may be due to the different behaviour of pregnant women in relation to the assimilation of information, or availability of it. Fan et al. (45) also associated higher COVID-19 knowledge with lower likelihood of depression, which allowed them to positively link fear with depression.

With regard to anxiety, the present study aimed at assessing the levels of anxiety in pregnant women and also the factors that may protect or predispose this population to anxiety during pregnancy. This was done given the consequences that such a psychological state may have for gestation in terms of threat of preterm birth, low birth weight, neurodevelopmental abnormalities, depression, nausea or vomiting during pregnancy, or low Apgar scores (39,54-56).

According to the reviewed literature, anxiety levels during the pandemic in pregnant women ranged from 45.9% (41) to 62% (30,36). Therefore, around two-thirds of pregnant women showed signs of anxiety, an increase of between 30% and 37% (57,58) in anxiety levels in relation to pre-pandemic studies. Despite the use of different tools in the measurement of anxiety levels, all of them reported higher scores than pre-pandemic studies (30, 32-38). Dagklis et al. (59), during confinement and using the Greek version of the State-Trait Anxiety Inventory, reported a prevalence of anxiety states of 34.2%, indicating a significant increase in anxiety levels during confinement (*p* < 0.001). At the same time, this study reported differences between the three trimesters of pregnancy and higher anxiety levels in the first week of quarantine, which gradually decreased in the second week and reached almost normal levels in the third week (59). Similar data were provided by the study conducted by Mappa et al. (44) in Italy days before the total closure decreed by the Italian government during March 2020. In both studies, there was a positive linear correlation between STAI-T and STAI-S, which may indicate that levels of anxiety in pregnancy are related to pre-existing levels of anxiety, in line with studies that determined an altered mental health status as the main risk factor for increased anxiety levels (44,60,61).

The work in Poland by Makara-Studzińska et al. (35) reported elevated anxiety levels in 52% of pregnant women and related the severity of anxiety to the time during the wave. In this regard, only the study in Brazil by Nomura et al. (51) reported different levels of anxiety in different regions, which, on the other hand, could be due to the different phases of the pandemic in a country as large as Brazil or to differences in financial status in a country with such economic inequality. Thus, Kajdy et al. (46) in an international multicentre study, identified financial status concern as a risk factor for high GAD-7 scores, but also identified a number of protective factors with lower GAD-7 scores, such as partner support, support from family members, older maternal age, or comfortable financial status.

Khamees et al. (38) in Egypt reported similar levels of anxiety between nulliparous and multiparous women, 45.27% vs. 47.28%. In this sense, there is controversy about the higher or lower levels of anxiety among nulliparous versus multiparous pregnant women. Some studies have found no statistically significant differences according to the number of pregnancies in the context of the COVID-19 pandemic. However, an association has been found between anxiety and educational level (36), which may be due to the greater awareness of older mothers of the threats of a disease with so much clinical variability. In relation to gestational age, contrary to what might be thought, the included studies have not shown statistically significant differences. The results in this sense have been contradictory in pre-pandemic periods, as some authors had reported higher levels in the first and third trimester (62), yet another study reported similar levels of anxiety (63).

Finally, there are a number of factors such as unplanned pregnancy, poor spousal support, negative behaviours, intolerance to the unknown, age, or financial status that act as risk factors for fear levels. In relation to anxiety, maternal age, social support, financial status, having a family member diagnosed with COVID-19, educational level, knowledge of COVID-19, or concern about maintaining prenatal consultations have been identified as risk factors for higher levels of anxiety.

Some of the limitations and weaknesses to be taken into account when interpreting the results of this study are as follows: very heterogeneous measurement tools were used, and all the included studies were cross-sectional; the characteristics of the population in terms of obstetrics (high or low risk) were not defined in the analysed studies; the lack of standardised scales for pregnant women during the 2020–2022 pandemic; and the samples were mostly collected online and in very heterogeneous periods from a pandemic context point of view.

### Conclusion

Based on the reviewed research, it can be concluded that the COVID-19 pandemic has had an impact on the mental health of pregnant women through increased levels of fear and anxiety. The analysed studies show a statistically significant relationship between levels of fear and anxiety in pregnant women during the pandemic.

Finally, based on the reviewed literature, it has not been possible to establish a relationship between significant factors such as gestational age, measures to control the health emergency, or difficulty of access to health services and high levels of fear or anxiety. There is a high degree of heterogeneity of risk factors, which points to the need for better observational research. In this sense, further research is needed to assess levels based on these factors in order to be able to define an association and try to avoid bias in the analyses. With the clear objective of minimising anxiety and fear in a period of special vulnerability, further study of predisposing or protective factors in contexts of special difficulty is needed.
